# Characteristics of morbidity and mortality conferences associated with the implementation of patient safety improvement initiatives, an observational study

**DOI:** 10.1186/s12913-016-1279-8

**Published:** 2016-01-30

**Authors:** Patrice François, Frédéric Prate, Gwenaëlle Vidal-Trecan, Jean-François Quaranta, José Labarere, Elodie Sellier

**Affiliations:** 1 Quality of Care Unit, University Hospital, and Research Unit, TIMC-IMAG (UMR 5525 CNRS/UJF-Grenoble 1), Grenoble, F-38043 France; 2 Public Health Department, University Hospital, Nice, F-06003 France; 3 Public Health Unit: Risk Management and Quality of Care, Paris Centre University Hospital Group, AP-HP, and Research Unit, (INSERM U738), Paris Descartes University, Sorbonne Paris cite, Paris, F- 75014 France

**Keywords:** Morbidity and mortality conferences, Patient safety, Quality improvement, Hospital

## Abstract

**Background:**

The aim of this study was to identify the characteristics of morbidity and mortality conferences (MMCs) associated with the implementation of patient health-care quality and safety improvement initiatives.

**Methods:**

We conducted an observational study of MMCs and followed up improvement initiatives for 1 year. Data on MMC baseline characteristics were abstracted using document analysis and observation of a meeting in three university hospitals in France (Grenoble, Nice, and Cochin [Paris] hospitals). Fifty-nine MMCs were included in medical (*n* = 24), surgical (*n* = 21), and anesthesiology and/or intensive care (*n* = 14) departments. An effectiveness index was computed by summing a composite score for each initiative pertaining to the MMC.

**Results:**

Overall, 282 initiatives were identified in 42 MMCs. During the follow-up period, 215 initiatives (76 %) were totally or partially implemented and the impact was evaluated for 73 (26 %). An effectiveness index higher than the median (i.e., ≥10) was associated with a standardized presentation of cases (81 % versus 29 %, *p* <0.001), recording of improvement initiatives (94 versus 57, *p* = 0.001), the existence of an annual activity report (94 % versus 68 %, *p* = 0.01), the prior dissemination of a meeting agenda (71 % versus 36 %, *p* = 0.007), longer meeting duration (109 versus 80 min, *p* = 0.005), anesthesiology and/or intensive care specialty (39 % versus 7 %, *p* = 0.02), a theme-focused MMC (29 % versus 4 %, *p* = 0.01), and a thorough analysis of failures (58 % versus 25 %, *p* = 0.01).

**Conclusions:**

This study suggests that the implementation of improvement initiatives relates to MCC characteristics. Recommendations for developing more effective patient safety-oriented MMCs can be proposed.

**Electronic supplementary material:**

The online version of this article (doi:10.1186/s12913-016-1279-8) contains supplementary material, which is available to authorized users.

## Background

Morbidity and mortality conferences (MMCs) were primarily established as an educational tool for surgeons in the United States [1]. Their use rapidly extended to other specialties and countries. MMCs seek to analyze medical errors and adverse events in order to improve medical practices [2–5]. Identifying and analyzing errors is basically an educational process and a training opportunity for residents [6, 7]. Furthermore, MMCs oriented toward clinical risk management can help develop the general competencies of those involved in practice-based learning and improvement as well as a system-based practice, mandated by the Accreditation Council for Graduate Medical Education (ACGME) [4, 7–12].

Evidence is lacking on whether the MMC is an effective tool for improving patient safety [12, 13]. Substantial variations exist in MMCs with regard to frequency, attendance, case selection or presentation, analysis methods, and follow-up [13, 14]. This observation may reflect the lack of explicit goals, methods, and format for MMCs [3, 4, 15]. However, it is not known whether the characteristics of MMCs are associated with effectiveness in healthcare quality and safety. Previous studies have reported conflicting results regarding the impact of MMCs. Few single-center studies found significant reductions in adverse events such as ventilator-associated pneumonia rates or cardiac arrest incidence [10, 16]. Other studies failed to show any improvement in patient clinical outcomes, [17, 18] probably due to the low incidence of adverse events [18].

Another way to assess the effectiveness of MMCs is to study their effect on care processes. Since changes in practices are mediated by the improvement initiatives that are decided on during the meetings, studying these initiatives may help assess the effectiveness of the conferences [8, 9, 11].

The aim of this multicenter study was to investigate the characteristics of MMCs and to find which of them were associated with the number of improvement initiatives and their implementation.

## Methods

### Study design

We conducted an observational study of MMC characteristics with a prospective follow-up of improvement initiatives in three university hospitals in France (Grenoble, Nice, and Cochin [Paris] hospitals). In each hospital, the organization of MMCs was defined in a guideline and departments could obtain methodological assistance from the quality-assurance team.

### Study sample

All MMCs established in the participating hospitals for more than 1 year were eligible. They were identified by researchers between September and December 2010. The purpose of the study was presented to the MMC leaders and their consent was required for participation in the study. All MMC leaders gave their informal oral consent for document analysis and requested the oral consent of MMC participants to accept the presence of two observers at a meeting. In case of refusal from the MMC leader and/or a participant, the MMC was not included in the study. Data collected on the forms contained no data directly or indirectly identifying patients or healthcare professionals. Ethics review board (Direction for clinical research, Grenoble university hospital) approval was not required for this observational study because no personal data was collected [19, 20].

### Data collection

In each center, data on MMC baseline characteristics were abstracted from document analysis by two researchers with the MMC leader present (Additional files 1 and 2). All MMC written documents produced during the year before the inclusion date were analyzed. These documents included the charter or the organizational procedure, all meeting reports, the annual activity reports, and all documents related to improvement initiatives decided in meetings. Data collected included department specialty, documents tracing the activity, number of meetings, number of cases presented, and number of senior physicians, residents, nurses and other paramedics who attended the meetings during the period under study. Attendance rates were computed by dividing the number of MMC attendants by the number of eligible professionals in the department.

Additionally, two researchers independently observed a meeting for each identified MMC, using a structured data collection form (Additional files 3 and 4). They recorded the types of participants, the format of presentations (use of slides, chronological presentation of facts, literature review), and the content of the discussion (investigation of adverse events and underlying factors). Observers attended two 4-h sessions together to standardize data collection and coding, and coding instructions were written in a data collection guide.

All improvement initiatives identified by direct observation or document analysis were followed up for 1 year. They were categorized according to the International Classification for Patient Safety [21]. At the end of the study period, MMC leaders were asked whether the initiatives had been implemented and if their impact had been evaluated. Two investigators independently reviewed the documents to ascertain whether the improvement initiatives were actually implemented and evaluated.

### Endpoint

The main endpoint of the study was an effectiveness index calculated for each department and based on the number and completion of improvement initiatives. Each single initiative was scored according to four items including designation of a person in charge of implementation (*yes* or *no*), definition of a timeline (*yes* or *no*), completion (*fully*, *partially*, or *not completed*), and evaluation of its impact (*fully*, *partially*, or *not evaluated*). Completion was categorized as *partially* when a part of the action plan was not achieved or when the entire target population was not reached. The evaluation was considered as *partial* if it was only an informal assessment and was considered as *complete* if the impact of the action was formally assessed by an indicator or audit. The initiative score was obtained by summing the points for each item (Table 1) and ranged from 0 to 6, with a higher score denoting higher levels of completion and evaluation.

**Table 1:** Items and scoring^a^ system for improvement initiative completion (*N* = 282)

	Points	Number	Percent
Designation of person in charge			
No	0	132	(46.8)
Yes	1	150	(53.2)
Definition of a timeline			
No	0	185	(65.6)
Yes	1	97	(34.4)
Completion of action			
None	0	67	(23.8)
Partial	1	28	(9.9)
Complete	2	187	(66.3)
Evaluation			
None	0	209	(74.1)
Partial	1	38	(13.5)
Complete	2	35	(12.4)

^a^For each improvement initiative, the score ranged from 0 to 6

The effectiveness index of MMCs within a department was obtained by summing the initiative scores related to this department. The effectiveness index was higher when the initiatives were more numerous and more thoroughly planned, implemented, and evaluated.

### Statistical analysis

MMC baseline characteristics were reported as numbers and percentages for categorical variables, and median and interquartile range (IQR) for continuous variables. The continuous variables, including the effectiveness index, were dichotomized according to their median. In univariate analysis, we examined the associations between the dichotomized effectiveness index and MMC characteristics using the chi-square test or Fischer’s exact test, when appropriate, for categorical variables, the chi-square test for trend for ordered categorical variables, and the Kruskal-Wallis test for continuous variables. We performed multivariate logistic regression analysis to estimate adjusted odds ratios for the characteristics that were independently associated with the dichotomized effectiveness index. Covariates were removed from a full non-parsimonious model using a backward approach with a *p*-value <0.10; *p*-values less than 0.05 were considered statistically significant. In case of multiple comparisons, the Bonferroni correction was applied to calculate a corrected threshold α_c_. Analyses were performed using Stata 11.0 (Stata Corp, College Station, TX, USA).

## Results

All solicited MMC leaders and participants agreed to participate in the study. The study sample consisted of 59 MMCs, including 24 (40.7 %) in medical units, 21 (35.6 %) in surgical units, and 14 (23.7 %) in anesthesiology or intensive care units. A median number of four meetings per MMC (IQR, 3–7) were conducted during the study period (Table 2). A median number of 18 cases were examined (IQR, 9–47), mostly deaths or complications from a medical procedure. Senior physicians and residents accounted for the vast majority of MMC attendants.

**Table 2:** Baseline morbidity and mortality conference characteristics abstracted from document analysis (*n* = 59)

Center, *n*, (%)		
Grenoble	29	(49.2)
Nice	21	(35.6)
Cochin	9	(15.3)
Specialty*, n*, (%)		
Medicine	24	(40.7)
Surgery	21	(35.6)
Anesthesiology and intensive care	14	(23.7)
Documents*, n*, (%)		
Written charter	53	(89.8)
Annual activity report	48	(81.4)
Nominative list of attendants	50	(84.7)
Meeting report for each meeting	53	(89.8)
Record of decided actions	45	(76.3)
Organization		
Number of meetings during the year, median, [IQR]	4	[3─7]
Number of cases studied during the year, median, [IQR]	18	[9─47]
Theme-focused MMC, n, (%)	10	(17.0)
Prior dissemination of meeting agenda, *n*, (%)	32	(54.2)
Professionals attending at least one meeting, %, [IQR]		
Senior physicians	80	[60─100]
Residents	77	[55─100]
Head nurses	67	[0─100]
Nurses	8	[0─19]

A meeting report was found for each meeting in 53 MMCs (90 %), whereas some meeting reports were lacking in five MMCs and no meeting report was found in one MMC. The improvement initiatives were recorded for 45 MMCs (76 %).

Direct observation of MMCs recorded 766 case presentations, including 352 deaths (45.8 %), 405 complications (52.7 %), and nine near-miss events. Senior physicians exclusively presented the cases in 34 MMCs (58 %) (Table 3). In the vast majority of MMCs, the clinical facts were described chronologically. In 13 MMCs (22 %), the presenter reported information drawn from a literature review. The discussion was centered on clinical practice (91 %) and less frequently on organizational issues (49 %). Defects in the patient’s care were sought in 54 MMCs (91 %) and were thoroughly analyzed in 25 MMCs (42 %) with a search for underlying factors. This cause analysis was based on a structured method in three MMCs (5 %). In eight MMCs (14 %), the previously identified initiatives were followed up at each subsequent meeting.

**Table 3:** Baseline morbidity and mortality conference characteristics recorded by direct observation (*n* = 59)

Characteristics	Number	Percent
Function of the professional presenting the cases, *n*, (%)		
Senior physician	34	(57.6)
Resident	4	(6.8)
Both	21	(35.6)
Cases presentation, *n*, (%)		
Standardized with use of slides	33	(55.9)
Chronological presentation of facts	54	(91.5)
Presentation of literature data	13	(22.0)
Topics of discussion, *n*, (%)		
Clinical practice	54	(91.5)
Communication/organizational issues	29	(49.2)
Failures, *n*, (%)		
Searched	54	(91.5)
Searched and thoroughly analyzed	25	(42.4)
Searched and analyzed using a structured method	3	(5.1)
Monitoring of previous initiatives, *n*, (%)	8	(13.6)
Length of meeting (min), median, [IQR]	87	[60─120]

A total of 282 improvement initiatives were identified in MMCs in 42 departments during the study period, with a median number of four initiatives per department (Table 4). Of these, 111 initiatives (39.4 %) targeted factors related to the staff, with 72 aiming at developing or amending checklists, protocols, or policies. Organizational, equipment, and patient factors accounted for 32.3, 8.5, and 7.1 % of improvement initiatives, respectively.

**Table 4:** Factors that contributed to an incident and were the target of an improvement initiative decided during morbidity and mortality conferences (*N* = 282)

Factors	Number	Percent
Staff	111	39.4
Training	32	11.3
Orientation	2	0.7
Supervision/assistance	4	1.4
Availability of checklists/protocols/policies	72	25.5
Adequate staff numbers/quality	1	0.4
Organizational/environmental	91	32.3
Matching physical environment to needs	8	2.8
Making arrangements for access to a service	17	6.0
Performing risk assessment/root cause analyses	2	0.7
Current code/specifications/regulations being met	9	3.2
Arranging ready access to protocols/policies/decision aids	31	11.0
Improved leadership/guidance	22	7.8
Matching of staff to tasks/skills	1	0.4
Improving safety culture	1	0.4
Other	36	12.8
Recommending a new practice	9	3.2
Literature review	14	5.0
Study of clinical cohorts	8	2.8
Improved file/traceability	3	1.1
Declaration to risk management unit	2	0.7
Agent/equipment	24	8.5
Provision of equipment	20	7.1
Regular audits	4	1.4
Patient	20	7.1
Provision of adequate care/support	7	2.5
Provision of patient education/training	6	2.1
Provision of protocols/decision aid	6	2.1
Provision of medication dispensing aid	1	0.4

The median score for each improvement initiative completion was 3 (IQR, 1–4). A person in charge was designated for 150 (53.2 %) actions and a timeline was defined for 97 (34.4 %) (Table 1). Overall, 215 initiatives (76.2 %) were implemented either partially or fully and their impact was evaluated for 73 (25.9 %).

The median effectiveness index per MMC was 10 (IQR, 3–43). In univariate analysis, the MMCs with an effectiveness index higher than the median were compared with those having a lower index than the median (Table 5). A higher effectiveness index was associated with a higher prevalence of documents, in particular with annual activity reports (*p* = 0.01) and with organizational issues: longer duration of meetings (*p* = 0.005), prior dissemination of a meeting agenda (*p* = 0.007), monitoring of previously decided actions (*p* = 0.04), and theme-focused MMCs (*p* = 0.01).

**Table 5:** Differences between the characteristics of morbidity and mortality conferences according to the level of the effectiveness index

	Index <10		Index ≥10		p*
	*N* = 28		*N* = 31		
Center, *n*, (%)					0.14
Grenoble	17	(60.7)	12	(38.7)	
Nice	9	(32.1)	12	(38.7)	
Paris	2	(7.1)	7	(22.6)	
Specialty, *n*, (%)					0.02
Medicine	14	(50.0)	10	(32.3)	
Surgery	12	(42.9)	9	(29.0)	
Anesthesia and intensive care	2	(7.1)	12	(38.7)	
Formalization characteristics*, n*, (%)					
Charter	23	(82.1)	30	(96.8)	0.08
Annual activity report	19	(67.9)	29	(93.5)	0.01
Nominative list of attendants	22	(78.6)	28	(90.3)	0.19
Meeting reports for each meeting	25	(89.3)	28	(90.3)	0.61
Record of decided actions	16	(57.1)	29	(93.5)	0.001
Organization					
No. of meetings during the year, median, [IQR]	4	[3–7]	4	[3–7]	0.65
No. of cases, median, [IQR]	19	[8–60]	15	[10–29]	0.63
Theme focused MCC*, n* (%)	1	(3.6)	9	(29.0)	0.01
Prior dissemination of meeting agenda, *n* (%)	10	(35.7)	22	(71.0)	0.007
Monitoring of previous actions, *n*, (%)	1	(3.6)	7	(22.6)	0.04
Length of meeting (min), median, [IQR]	80	[50–90]	118	[60–120]	0.005
No. of professionals attending, %, [IQR]					
Physicians, % [IQR]	82	[59–100]	80	[68–100]	0.83
Residents, % [IQR]	67	[60–100]	78	[50–100]	0.36
Head nurses, % [IQR]	60	[0–100]	75	[0–100]	0.79
Nurses, % [IQR]	2	[0–12]	9	[3–21]	0.06
Cases presentation and discussion					
Standardized with use of visual support, *n* (%)	8	(28.6)	25	(80.6)	<0.001
Presentation of literature data, *n* (%)	1	(3.6)	12	(38.7)	0.001
Search of failures, *n*(%)	24	(85.7)	30	(96.8)	0.15
Thorough analysis of failure, *n*,(%)	7	(25.0)	18	(58.1)	0.01

Abbreviations: *IQR* interquartile range, *MMC* morbidity and mortality conference

*p-values must be interpreted with respect to the threshold αc = 0.002, adjusted by the Bonferroni method

The higher effectiveness index was also associated with meeting characteristics: when the presentation of cases was standardized with the use of visual aids (*p* < 0.001), when the presentation included literature data (*p* = 0.001), when failures were sought and thoroughly analyzed with the search for causes of adverse events (*p* = 0.01). When interpreting *p*-values with the threshold α_c_ = 0.002, the only features associated with the effectiveness of MMCs were standardized presentation using visual aids and recording of decisions for improvement initiatives.

In multivariate analysis, a standardized presentation using visual aids (adjusted odds ratio = 2.89, 95 % confidence interval, [1.66–5.04]) and recording of decisions for improvement initiatives (aOR = 3.31, 95 % CI, [1.54–7.1]) remained independently associated with a high effectiveness index.

## Discussion

This study indicates that MMCs may produce initiatives for improving the quality and safety of care and that most of these initiatives are actually implemented. However, this ability to initiate improvement actions varies widely across MMCs.

We found that the effectiveness of MMCs, as reflected by a composite index, was associated with the standardized presentation of cases using visual aids. This finding was consistent with previous studies reporting that the use of a standard format or slides for case presentations enhanced the effectiveness of MMCs in terms of participant satisfaction and production of improvement initiatives [22, 23].

Other MMC baseline characteristics associated with improvement initiatives were formalization and organizational issues: production of annual activity reports, prior dissemination of a meeting agenda, recording of improvement initiatives, longer meetings, and theme-focused meetings. These characteristics imply a more formalized organization and a higher level of traceability. This effort to formalize the meetings reflects the importance given to this activity as well as a high level of organizational culture consistent with the principles of quality improvement [24]. The association of anesthesiology and intensive care departments with a high effectiveness index is not surprising because anesthesiology is recognized as one of the safest clinical specialties and a leader in patient safety [25].

This study fails to show a significant association between attendance of healthcare professionals and the effectiveness index of MMCs. However, many studies have found that multidisciplinary MMCs were more oriented toward the analysis of systemic causes of adverse events and were more likely to implement improvement initiatives [9, 12, 26]. In MMCs attended only by physicians, the debate tends to focus on clinical questions and medical practices, whereas in multidisciplinary MMCs, organizational issues are more often discussed [27]. Eliciting input from all staff involved in patient care has been described as essential to a high-quality investigation because the patient’s clinical course is thoroughly overviewed [13, 18, 28]. A multidisciplinary approach might also foster a culture of teamwork [1–3, 9, 11]. Above all, MMCs are a direct means to involve staff in quality-improvement initiatives [10].

The depth of the analysis of adverse events appears to be a key issue for MMC effectiveness. The thoroughness of the analysis of root causes is contingent upon the use of a method, time availability, and the involvement of all stakeholders. This is rarely compatible with the format of the traditional MMC. We observed that only three MMCs used a structured method. This observation was in accordance with Aboumatar et al., who reported that only one team used a structured method for incident analysis in 12 MMCs at Johns Hopkins Hospital [13].

In experiments intended to guide MMCs toward patient safety, one of the main changes is to facilitate root cause analysis of events in order to promote system changes [11, 12]. Most often, this is done using a formal method of analysis derived from Ichikawa’s fishbone diagram [4, 11, 26] or from the protocol of the Association of Litigation and Risk Management (ALARM) [10, 12, 29]. In many cases, the investigation was conducted outside and before the meeting in which the case was discussed [4, 10, 12, 23]. Depending on the study, the case selected for analysis was assigned to a resident or fellow for investigation within a framework called a “systems audit,” [4] “learning from the defect tool,” [12] “audit-based program,” [10] or “assessment tool.” [23] The time spent on this task is estimated at 35 h [4]. The externalization of adverse event investigation, before or after the meeting, makes it possible to devote more time to analysis and to meet all stakeholders involved in the event who might not participate in the MMC. Associating the effectiveness index with the organization of thematic MMCs is also part of a detailed analysis. In these meetings, the cases selected refer to the same issues. A more focused discussion facilitates a more thorough analysis of adverse events [6].

Certain authors noted that MMCs were missed opportunities for exploring systems contributing to medical errors and adverse outcomes in patient care and for implementing system changes [4, 5, 11]. System-based issues are rarely identified in MMCs and the time devoted to discussing interventions to improve patient care is insufficient [5]. Various experiments, usually conducted at a single hospital or department, have been designed to change the format of MMCs to move toward patient safety and enhancing residents’ ACGME competency in systems-based practice. Most of these safety-oriented MMCs include the aforementioned characteristics (i.e., multidisciplinary participation, standardized presentation, and root cause analysis).

Other frequently reported changes relate to criteria for selecting cases and monitoring improvement initiatives. The number and types of cases presented during MMCs are extremely variable and affect the content of discussions and outcomes of the MMC [5, 27]. Some authors advocate a rapid analysis of all deaths and complications because unexamined cases are missed opportunities to identify failures [30, 31]. Others consider a detailed investigation of a limited number of adverse events to be more effective to mitigating recurrent errors than a superficial investigation of a large number [6, 12, 13]. Our study is not contributive to this debate because we did not find any association between the number of cases examined by the MMC and the MMC’ index of effectiveness. In traditional MMCs, a large proportion of the cases presented were selected for their educational interest or potential clinical teaching value and often lack root cause analysis [11, 14, 17]. To guide MMCs toward patient safety, one study recommended a change consisting in selecting the cases on the basis of the potential for highlighting important healthcare system safety issues [8]. This selection is usually made by a multidisciplinary committee or a mortality review task force [9–11]. Planning and monitoring the implementation of improvement initiatives are other major issues. Depending on the context, this task is usually entrusted to specific working groups, an executive board, a quality-improvement committee, or a performance improvement meeting [9, 11, 12, 26].

The present study’s results, as well as published evidence, do not clearly identify an ideal format for MMCs. However, we can formulate a number of recommendations for care safety-oriented MMCs:

- Clearly defining the goals of the MMC and its functioning in a charter;
- Formalizing and tracking the activity of MMCs in meeting and annual reports;
- Using a standardized format and visual aids for case presentation;
- Analyzing the root causes of failures, using a structured method;
- Organizing the follow-up of actions for improvement;
- Inviting all staff to participate in multidisciplinary meetings;
- Selecting the cases presented based on their potential for systemic improvement.

This study had several limitations. First, the endpoint of MMC effectiveness was not an objective measure of improvement but an ad hoc index based on decision making and completion of initiatives for improvement. Taking action does not guarantee the effectiveness of the action but taking action is a step that can lead to improvement. We therefore considered, as other authors have, that the completion of improvement initiatives could be a reasonable surrogate endpoint for assessing the effectiveness of MMCs [8, 9, 11].

Second, the results from our multivariable logistic regression analysis should be interpreted with caution because the relatively limited number of observations compared with the large number of independent variables may lead to overfitting. Colinearity between independent variables might explain why unadjusted associations did not remain significant in multivariable analysis.

Third, this study was conducted in three university hospitals in France wherein the development of MMCs is rather recent (i.e., dating from the 1990s) and the participation in MMCs is not required in the medical training curriculum. Hence, these findings may not apply to other settings or countries.

## Conclusions

This study demonstrates substantial variations in MMC characteristics, which may relate to their effectiveness in improving patient safety. The framework of MMCs is highly flexible, which is a cause of MMC variability. However, this flexibility enables each team to adapt the MMC format to its objectives and constraints, which may contribute to the involvement of physicians. It is tempting to rely on MMCs to involve healthcare providers in patient safety management, but this goal requires revising the functioning of MMCs to establish a more rigorous methodological framework and to guide MMCs toward the identification, analysis, and prevention of adverse events.

## Additional files

Additional file 1: **Analyse documentaire.** (DOC 105 kb)

Additional file 2: **Document analysis.** (DOCX 22 kb)

Additional file 3: **Observation d'une réunion.** (DOC 81 kb)

Additional file 4: **Observation of MMC meeting.** (DOCX 23 kb)
