# Supplementary material for: Characteristics of morbidity and mortality conferences associated with the implementation of patient safety improvement initiatives, an observational study
Source: BMC Health Serv Res. 2016 Jan 30;16:35. doi: 10.1186/s12913-016-1279-8 (PMC4734851; doi:10.1186/s12913-016-1279-8)
Supplement: Supplementary file 1 — Analyse documentaire. (DOC 105 kb) [file 12913_2016_1279_MOESM1_ESM.doc]

**Analyse documentaire**

| **Investigateur :**…………………… | | | | | **Date :**…………... | | | | **Identifiant RMM** :………... | |
| --- | --- | --- | --- | --- | --- | --- | --- | --- | --- | --- |
| **Etablissement :**……………………………. | | | | | | | **Secteur :** Pôle…………….…………..  Service………………………  Unité………………………… | | | |
| **Spécialité :**  Médecine⁭ | | | Chirurgie⁭ | | | Réanimation⁭ | | Obstétrique⁭ | | Urgences ⁭ |
| Psychiatrie⁭ | Anesthésie⁭ | | Pédiatrie⁭ | | | SSR⁭ | | SLD⁭ | | HAD⁭ |
| **Année étudiée** : | | De ____/_______ à ____/______ (mm_/_aa) | | | | | | | | |
| **Date de mise en place de la RMM** | | | | ____/_______ (mm_/_aa) | | | | | | |

| **Existe-t-il une charte de la RMM ?** ( procédure, règlement intérieur) | Oui⁭ | Non⁭ |
| --- | --- | --- |

Si existante, la charte contient: *(Coter la réponse de 0 = absente à 3 = très détaillée)*

|  | **0** | **1** | **2** | **3** |
| --- | --- | --- | --- | --- |
| La définition des objectifs | ⁭ | ⁭ | ⁭ | ⁭ |
| La description de la périodicité des réunions et de la durée | ⁭ | ⁭ | ⁭ | ⁭ |
| La description des modalités de sélection des cas | ⁭ | ⁭ | ⁭ | ⁭ |
| La description des critères de sélection des cas | ⁭ | ⁭ | ⁭ | ⁭ |
| La description des modalités de suivi des actions | ⁭ | ⁭ | ⁭ | ⁭ |

| **Existe-t-il un bilan annuel?** | Oui⁭ | Non⁭ |
| --- | --- | --- |

Si existant, ce bilan annuel contient :

| Le nombre de RMMs dans l’année | Oui⁭ | Non⁭ |
| --- | --- | --- |
| Le nombre de nombre de cas analysés dans l’année | Oui⁭ | Non⁭ |

| *(Coter la réponse de 0 = absente à 3 = très détaillée)* | **0** | **1** | **2** | **3** |
| --- | --- | --- | --- | --- |
| La liste des actions mises en œuvre dans l’année | ⁭ | ⁭ | ⁭ | ⁭ |
| La liste des thèmes/problèmes discutés dans l’année | ⁭ | ⁭ | ⁭ | ⁭ |

# Comptes Rendus de réunions (CR)

Nombre de réunions dans l’année : ______ Nombre de CR rédigés dans l’année : ______

Si existants, les CR contiennent:

| La liste nominative des personnes présentes | Oui⁭ | Non⁭ |
| --- | --- | --- |

*Si oui, remplir le tableau joint*

| *(Coter la réponse de 0 = absente à 3 = très détaillée)* | **0** | **1** | **2** | **3** |
| --- | --- | --- | --- | --- |
| Le suivi des actions décidées en RMMs antérieures | ⁭ | ⁭ | ⁭ | ⁭ |
| Le résumé de chaque cas | ⁭ | ⁭ | ⁭ | ⁭ |
| La description des thèmes débattus, des problèmes soulevés | ⁭ | ⁭ | ⁭ | ⁭ |
| La trace des actions décidées | ⁭ | ⁭ | ⁭ | ⁭ |

| *(Coter la réponse de 0 =* Jamais *à 3 =* Toujours*)* | **0** | **1** | **2** | **3** |
| --- | --- | --- | --- | --- |
| L’anonymat  des patients est il respecté dans les CR? | ⁭ | ⁭ | ⁭ | ⁭ |

| Nombre de dossiers | Décès____ | Complications____ | EPR____ | Total____ |
| --- | --- | --- | --- | --- |
|  | Présentation de séries cliniques ____ | | |  |

Traçabilité des réunions (noter les particularités) : __________________________________

___________________________________________________________________________

___________________________________________________________________________

Nombre de thèmes ayant fait l’objet d’un débat en réunion sans

décision d’action

Dont : Questions de pratique clinique, (décision médicale) ___

Questions de pratique de soins paramédicaux ___

Question d’organisation des soins, de l’équipe ___

Citer 5 thèmes significatifs :

|  |
| --- |
|  |
|  |
|  |
|  |

Nombre de thèmes ayant fait l’objet d’une proposition d’action ___

*(1 fiche de suivi de l’action doit être renseignée pour chaque action)*

# Modalités de fonctionnement de la RMM

**Participation aux réunions:**

Nombre de médecins ayant participé à au moins 1 RMM dans l’année __

Nombre de médecins ayant participé à au moins la moitié des RMM de l’année __

*Nombre de médecins exerçant dans le secteur* __

Nombre d’internes ayant participé à au moins 1 RMM dans l’année __

*Nombre d’internes exerçant dans le secteur* __

Nombre de cadres de santé ayant participé à au moins 1 RMM dans l’année __

*Nombre de cadres de santé exerçant dans le secteur* __

Nombre d’infirmières ayant participé à au moins 1 RMM dans l’année __

*Nombre d’infirmières exerçant dans le secteur* __

Nombre de personnes extérieures ayant participé à au moins 1 RMM dans l’année __

**Planification des RMMs**

Il existe un calendrier prévisionnel des réunions Oui⁭ Non⁭

Les réunions ont lieu à un jour et un horaire fixe : Oui⁭ Non⁭

Autre planification préciser : ________________________________

Un ordre du jour de la réunion est diffusé à l’avance : Oui⁭ Non⁭

**Critères de sélection des cas**

Patients décédés : Tous⁭ Certains⁭ Aucun⁭

Complications : Oui, selon critères définis⁭ Oui, sans critères⁭ Non⁭

EPR : Oui, selon critères définis⁭ Oui, sans critères⁭ Non⁭

**Existe-t-il des RMM thématiques** : Oui⁭ Non⁭ ; si oui combien dans l’année __

**Préciser les modalités de sélection des cas (qui, quand, comment)**

…………………………………………………………………………………………………

…………………………………………………………………………………………………

………………………………………………………………………………………………………………………………………………………………………………………………………………………………………………………………………………………………………

**Liste nominative des participants aux RMMs de l’année**

| **Nom, Prénom** | **N°Q** | **Grade*** | **Fonction^#^** | **Service** | **Nombre de participations sur l’année** |
| --- | --- | --- | --- | --- | --- |
|  |  |  |  |  |  |
|  |  |  |  |  |  |
|  |  |  |  |  |  |
|  |  |  |  |  |  |
|  |  |  |  |  |  |
|  |  |  |  |  |  |
|  |  |  |  |  |  |
|  |  |  |  |  |  |
|  |  |  |  |  |  |
|  |  |  |  |  |  |
|  |  |  |  |  |  |
|  |  |  |  |  |  |
|  |  |  |  |  |  |
|  |  |  |  |  |  |
|  |  |  |  |  |  |
|  |  |  |  |  |  |
|  |  |  |  |  |  |
|  |  |  |  |  |  |
|  |  |  |  |  |  |
|  |  |  |  |  |  |
|  |  |  |  |  |  |
|  |  |  |  |  |  |
|  |  |  |  |  |  |
|  |  |  |  |  |  |

* Grade = PUPH, MCU-PH, CCA, PH etc… ;

# Fonction : chef de service, chef de pôle, responsable d’UF, animateur de la RMM
