# Supplementary material for: Characteristics of morbidity and mortality conferences associated with the implementation of patient safety improvement initiatives, an observational study
Source: BMC Health Serv Res. 2016 Jan 30;16:35. doi: 10.1186/s12913-016-1279-8 (PMC4734851; doi:10.1186/s12913-016-1279-8)
Supplement: Supplementary file 2 — Document analysis. (DOCX 22 kb) [file 12913_2016_1279_MOESM2_ESM.docx]

**Document analysis**

Investigator :…………………… Date :…………... MMC identifier :………

Hospital :……………………………. Sector : Division…………….……

Department………………

Unit……………………

**Specialty** : Medicine⁭ Surgery ⁭ intensive care⁭ Obstetrics⁭ Emergency room

Psychiatrie Anesthesiology Pediatrics Rehabilitation Geriatrics Home hospitalization

Year studied: from ____/_______ to ____/______ (mm_/_yy)

Date of MMC’ creation ____/_______ (mm_/_yy)

**Is there a MMC charter ?** (Procedure, internal rules) Yes No

If existing, the charter contains *(Rate response from 0 = absent to 3 = very detailed)*

|  | **0** | **1** | **2** | **3** |
| --- | --- | --- | --- | --- |
| The definition of objectives | ⁭ | ⁭ | ⁭ | ⁭ |
| The description of meetings periodicity and duration | ⁭ | ⁭ | ⁭ | ⁭ |
| The description of rules for cases selection | ⁭ | ⁭ | ⁭ | ⁭ |
| The description of criteria for cases selection | ⁭ | ⁭ | ⁭ | ⁭ |
| The description of the initiatives monitoring | ⁭ | ⁭ | ⁭ | ⁭ |

**Is there an annual report?** Yes No

If existing, the charter contains:

- The number of meetings in the year Yes No
- The number of cases analyzed in the year Yes No

| *(Rate response from 0 = absent to 3 = very detailed)* | **0** | **1** | **2** | **3** |
| --- | --- | --- | --- | --- |
| - The list of initiatives implemented in the year |  | ⁭ | ⁭ | ⁭ |
| - The list of topics / issues discussed in the year |  |  |  |  |

**Meeting reports (MR)**

Number of meetings during the year: ______ Number of MR drafted in the year: ______

If existing, the MR contains:

- The list of attendees Yes No

| *(Rate response from 0 = absent to 3 = very detailed)* | **0** | **1** | **2** | **3** |
| --- | --- | --- | --- | --- |
| - The follow-up of initiatives decided in past MMCs |  | ⁭ | ⁭ | ⁭ |
| - The description of the topics and issues discussed |  |  |  |  |
| - A record of agreed initiatives |  |  |  |  |

| *(Rate response from 0 = absent to 3 = very detailed)* | **0** | **1** | **2** | **3** |
| --- | --- | --- | --- | --- |
| - The MR respect the anonymity of patients |  | ⁭ | ⁭ | ⁭ |

Number of patient records: Deaths ____ Complications ____ Near misses ______

Clinical series ____

Meetings traceability (note the peculiarities)_________________________________________

____________________________________________________________________________

**Number of discussed topics without acting decision of which:**

- Clinical practice issues (medical decision) ____
- Paramedical practice issues ____
- Care organization issues ____

Cite 5 significant topics: ________________________________________________________

_____________________________________________________________________________

**Number of topics with proposed initiative:** ___

*(1 action tracking form must be filled for each action)*

**Operating modes of the MMC**

**Meeting attendance:**

- Number of physicians who participated in at least one MMC in the year ___
- Number of physicians who participated in half of MMC, or more, in the year ___
- *Number of doctors in the sector* ___
- Number of medical residents who participated in at least one MMC in the year ___
- *Number of medical residents in the sector* ___
- Number of head nurses who participated in at least one MMC in the year ___
- *Number of head nurses in the sector* ___
- Number of nurses who participated in at least one MMC in the year ___
- *Number of nurses in the sector* ___
- Number of external professionals who participated in at least one MMC in the year ___

**Planning of MMCs**

- There is a projected schedule of meetings Yes No
- Meetings are held at a fixed date and schedule Yes No

Other planning, *specify*: ____________________________________________________

An agenda of the meeting was spread in advance Yes No

**Criteria for selection of cases**

Dead patients: all some none

Complications: Yes with criteria Yes without criteria None

Near misses: Yes with criteria Yes without criteria None

**Are there any theme focused MMC?** Yes No ; If so how many in the year: ___

Specify the methods for selecting cases (who, when, how): ____________

____________________________________________________________

**Nominative list of participants to meetings in the year**

| **name** | **N°** | **Grade** | **Function^#^** | **Department** | **Number of participations** |
| --- | --- | --- | --- | --- | --- |
|  |  |  |  |  |  |
|  |  |  |  |  |  |
|  |  |  |  |  |  |
|  |  |  |  |  |  |
