# Supplementary material for: Characteristics of morbidity and mortality conferences associated with the implementation of patient safety improvement initiatives, an observational study
Source: BMC Health Serv Res. 2016 Jan 30;16:35. doi: 10.1186/s12913-016-1279-8 (PMC4734851; doi:10.1186/s12913-016-1279-8)
Supplement: Supplementary file 3 — Observation d'une réunion. (DOC 81 kb) [file 12913_2016_1279_MOESM3_ESM.doc]

**Observation de RMM**

| **Investigateur :**…………………… | | **Date :**…………... | | **Identifiant RMM** :………... |
| --- | --- | --- | --- | --- |
| **Durée de la réunion** | …………minutes | |  | |

**Structure de la RMM**

Participants

| **Appartenant au service concerné par la RMM** | | | | | Total ______ |
| --- | --- | --- | --- | --- | --- |
| Médecin______ | Interne______ | Cadre de santé______ | IDE______ | Autres______ | |

Si autres, préciser :…………………………………………………………………………………………………

Pour les services de chirurgie préciser la présence des anesthésistes :…………………………………………….

| **Appartenant à un autre service de l’établissement** | Total ______ |
| --- | --- |

Préciser les statuts…..:………………………………………………………..……………….

| **Extérieur à l’établissement** | Total ______ |
| --- | --- |

Préciser les statuts…..:………………………………………………………..……………….

| **Animateur identifié** | Oui⁭ | Non⁭ | Si oui, préciser le statut ………………… |
| --- | --- | --- | --- |
| **Secrétaire de séance identifiée** | Oui⁭ | Non⁭ | Si oui, préciser le statut …………………. |

**Contenu de la RMM**

| Suivi des actions antérieures | Oui⁭ | Non⁭ |
| --- | --- | --- |

Si oui préciser:………………………………………………………………….......................

| Nombre de dossiers | Décès____ | Complications____ | EPR____ | Total____ |
| --- | --- | --- | --- | --- |
|  | Présentation de séries cliniques ____ | | |  |

**Présentation**

**-Présentateur :**

|  | Pour tous les cas | Souvent | Parfois | Jamais |
| --- | --- | --- | --- | --- |
| Médecin | □ | □ | □ | □ |
| Interne | □ | □ | □ | □ |
| Autre | □ | □ | □ | □ |

Si autre, préciser …………………………………………………………………………………………………………………………….

**-**Modalités

|  | Pour tous les cas | Souvent | Parfois | Jamais |
| --- | --- | --- | --- | --- |
| Utilisation d’un support visuel | □ | □ | □ | □ |
| Utilisation (ou disponibilité) de documents médicaux (dossiers, imagerie, biologie..) | □ | □ | □ | □ |
| Présentation factuelle et chronologique | □ | □ | □ | □ |
| Présentation interprétative/analytique (l’exposé oriente vers la question à débattre): | □ | □ | □ | □ |
| Présentation « documentée » (l’exposé apporte des éléments de la littérature ou RPC) | □ | □ | □ | □ |

**Discussion**

**Caractériser l’organisation de la discussion**

| Tour de table⁭ | Distribution de la parole/Questionnement⁭ | Aucune⁭ |
| --- | --- | --- |

Commentaire :…………………………………………………………………………………...

**Caractériser la participation des différents professionnels aux débats**

|  | Tous | Majorité | Minorité | Aucun | NA |
| --- | --- | --- | --- | --- | --- |
| Médecin | ⁭ | ⁭ | ⁭ | ⁭ | ⁭ |
| Interne | ⁭ | ⁭ | ⁭ | ⁭ | ⁭ |
| Cadre | ⁭ | ⁭ | ⁭ | ⁭ | ⁭ |
| IDE | ⁭ | ⁭ | ⁭ | ⁭ | ⁭ |
| Autres | ⁭ | ⁭ | ⁭ | ⁭ | ⁭ |

**Décrire le contenu et les issues de la discussion**

|  | Pour tous les cas | Souvent | Parfois | Jamais |
| --- | --- | --- | --- | --- |
| Le débat centré sur des pratiques médicales (pertinence, décision alternative, erreur…) | □ | □ | □ | □ |
| Le débat centré sur des problèmes de communication/d’organisation | □ | □ | □ | □ |
| Le débat évoque des questions de procédure de « récupération » ou de barrière de sécurité | □ | □ | □ | □ |

Commentaire :…………………………………………………………………………………………………………………………………………………………………………………….

| **Les dysfonctionnements sont recensés:** | Oui⁭ | Non⁭ |
| --- | --- | --- |

Si oui :

Recherche superficielle □ Recherche approfondie□ Utilisation d’une méthode□

Préciser :………………………………………………………………………………………………………..

| **Les causes expliquant les dysfonctionnements identifiés sont recherchées :** | Oui⁭ | Non⁭ |
| --- | --- | --- |

Si oui, il s’agit d’une recherche :

| Très superficielle□ | Superficielle□ | Approfondie□ | Très approfondie□ |
| --- | --- | --- | --- |

| Une méthode formelle d’analyse est utilisée | Oui⁭ | Non⁭ |
| --- | --- | --- |

Préciser :………………………………………………………………………………………………………..

Actions

|  | Pour tous les cas | Souvent | Parfois | Jamais |
| --- | --- | --- | --- | --- |
| Des actions correctrices sont décidées si des dysfonctionnements sont identifiés | □ | □ | □ | □ |
| Un responsable est désigné pour l’action | □ | □ | □ | □ |
| Un échéancier est fixé pour l’action | □ | □ | □ | □ |

**Perceptions de l’observateur**

| L’ambiance est apparue conviviale, détendue | Pas du tout d’accord□ | Plutôt pas d’accord□ | Plutôt d’accord□ | Tout à fait d’accord□ |
| --- | --- | --- | --- | --- |
| Les participants sont parvenus à éviter de blâmer (critiquer) un professionnel ayant commis une erreur | Pas du tout d’accord□ | Plutôt pas d’accord□ | Plutôt d’accord□ | Tout à fait d’accord□ |

Estimation du temps consacré : aux présentations ____%

Aux discussions ____%

Au suivi des plan d’action ____%

Commentaires de l’observateur : points particuliers, aspects positifs ou négatifs :

………………………………………………………………………………………………….………………………………………………………………………………………………….………………………………………………………………………………………………….………………………………………………………………………………………………….………………………………………………………………………………………………….………………………………………………………………………………………………….………………………………………………………………………………………………….………………………………………………………………………………………………….………………………………………………………………………………………………….………………………………………………………………………………………………….………………………………………………………………………………………………….………………………………………………………………………………………………….………………………………………………………………………………………………….………………………………………………………………………………………………….………………………………………………………………………………………………….………………………………………………………………………………………………….………………………………………………………………………………………………….………………………………………………………………………………………………….………………………………………………………………………………………………….………………………………………………………………………………………………….………………………………………………………………………………………………….………………………………………………………………………………………………….………………………………………………………………………………………………….………………………………………………………………………………………………….………………………………………………………………………………………………….………………………………………………………………………………………………….………………………………………………………………………………………………….………………………………………………………………………………………………….………………………………………………………………………………………………….………………………………………………………………………………………………….………………………………………………………………………………………………….………………………………………………………………………………………………….………………………………………………………………………………………………….…………………………………………………………………………………………………
