# Supplementary material for: Characteristics of morbidity and mortality conferences associated with the implementation of patient safety improvement initiatives, an observational study
Source: BMC Health Serv Res. 2016 Jan 30;16:35. doi: 10.1186/s12913-016-1279-8 (PMC4734851; doi:10.1186/s12913-016-1279-8)
Supplement: Supplementary file 4 — Observation of MMC meeting. (DOCX 23 kb) [file 12913_2016_1279_MOESM4_ESM.docx]

**Observation of MMC**

Investigator :…………………… Date :…………... MMC identifier :………

Duration of the meeting: …….. minutes

**MMC composition.**

**Participants**

Professionals working in the concerned department: total …...

Physicians …..., Medical residents …..., Head nurses …..., Nurses …..., Others …...

*If others, specify*: …………………………………………………………………….

Professionals working in other department: total …....

*Specify the professional statutes*: ……………………………………………………..

Professionals external to the hospital: total …....

*Specify the professional statutes*: …………………………………………………….

**Identified moderator:** Yes No, If so specify the statute: …………………………………

**Identified session secretary:** Yes No, If so specify the statute: …………………………

**MMC contents**

Monitoring of previous actions: Yes No, If so specify: …………………………………

Number of patient records: Deaths …..., Complications …..., Near misses …..., total …....

Clinical series …...

**Case presentations**

Presenter

|  | All cases | Often | Sometimes | Never |
| --- | --- | --- | --- | --- |
| Physician | □ | □ | □ | □ |
| Medical resident | □ | □ | □ | □ |
| Other | □ | □ | □ | □ |

*If other, specify:* ………………………………………………………………………

Methods of presentation

|  | All cases | Often | Sometimes | Never |
| --- | --- | --- | --- | --- |
| Using a visual support | □ | □ | □ | □ |
| Using patient medical record | □ | □ | □ | □ |
| Interpretive / analytical presentation | □ | □ | □ | □ |
| Presentation documented with literature data | □ | □ | □ | □ |

**Discussion**

Characterize the organization of the discussion

Round □, Distribution of speech □, no organization □

Comment : …………………………………………………………………………………….

**Characterize the participation of different professionals to the debates**

|  | All | Majority | Minority | None | NA* |
| --- | --- | --- | --- | --- | --- |
| Physicians | □ | □ | □ | □ | □ |
| residents | □ | □ | □ | □ | □ |
| Head nurses | □ | □ | □ | □ | □ |
| Nurses | □ | □ | □ | □ | □ |
| Others | □ | □ | □ | □ | □ |

*not applicable

**Describe the content and outcome of the discussion**

|  | All cases | Often | Sometimes | Never |
| --- | --- | --- | --- | --- |
| The debate focused on medical practices | □ | □ | □ | □ |
| The debate focused on communication or organization problems | □ | □ | □ | □ |
| The debate mentions issues of recovery process or safety barrier | □ | □ | □ | □ |

Commentary: ……………………………………………………………………………………

**Failures are identified:** Yes No

If so, Superficial analysis □, Thorough analysis □ , Using a method □

If so, specify: ……………………………………………………………………………..

**The root causes of the identified failures are sought:** Yes No

If so, this search is: Very superficial □, Superficial □, Thorough □, Very thorough□

If so, using a method: Yes No

Specify: …………………………………………………………………………………..

**Improvement initiatives**

|  | All cases | Often | Sometimes | Never |
| --- | --- | --- | --- | --- |
| Corrective actions are decided when failures are identified | □ | □ | □ | □ |
| A person in charge of the action is designated | □ | □ | □ | □ |
| A deadline is set for action | □ | □ | □ | □ |

**Perceptions of the observer**

|  | Totally disagree | Rather disagree | Rather agree | Totally agree |
| --- | --- | --- | --- | --- |
| The ambiance appeared friendly, relaxed | □ | □ | □ | □ |
| A person in charge of the action is designated | □ | □ | □ | □ |
| Participants were able to avoid blaming a professional who committed an error | □ | □ | □ | □ |

Estimating the time devoted to: Case presentations ….. minutes

Discussion ….. minutes

Monitoring of previous actions ….. minutes

Comments of the observer: Special Topics, positive and negative aspects:

…………………………………………………………………………………………………………………………………………………………………………………………………………………………………………………………………………………………………………………………………………………………………………………………………………………………………………………………………………………………………………………………………………………………………………………………………………………………………………………………………………………………………………………
